# Anti-Huntington’s Effect of Rosiridin via Oxidative Stress/AchE Inhibition and Modulation of Succinate Dehydrogenase, Nitrite, and BDNF Levels against 3-Nitropropionic Acid in Rodents

**DOI:** 10.3390/biom12081023

**Published:** 2022-07-23

**Authors:** Muhammad Afzal, Nadeem Sayyed, Khalid Saad Alharbi, Sami I. Alzarea, Mohammed Salem Alshammari, Fadhel A. Alomar, Sattam Khulaif Alenezi, Anwarulabedin Mohsin Quazi, Abdulaziz I. Alzarea, Imran Kazmi

**Affiliations:** 1 Department of Pharmacology, College of Pharmacy, Jouf University, Sakaka 72341, Saudi Arabia; kssalharbi@ju.edu.sa (K.S.A.); samisz@ju.edu.sa (S.I.A.); amquazi@ju.edu.sa (A.M.Q.); 2 School of Pharmacy, Glocal University, Saharanpur 247121, India; snadeem.pharma@gmail.com; 3 Department of Pharmacy Practice, Unaizah College of Pharmacy, Qassim University, Buraydah 52571, Saudi Arabia; m.alshammari@qu.edu.sa; 4 Department of Pharmacology and Toxicology, College of Clinical Pharmacy, Imam Abdulrahman Bin Faisal University, P.O. Box 1982, Dammam 31441, Saudi Arabia; falomar@iau.edu.sa; 5 Department of Pharmacology and Toxicology, Unaizah College of Pharmacy, Qassim University, Buraydah 52571, Saudi Arabia; sk.alenezi@qu.edu.sa; 6 Department of Clinical Pharmacy, College of Pharmacy, Jouf University, Sakaka 72341, Saudi Arabia; aialzarea@ju.edu.sa; 7 Department of Biochemistry, Faculty of Science, King Abdulaziz University, Jeddah 21589, Saudi Arabia

**Keywords:** Huntington’s disease, 3-nitropropionic acid, rosiridin, neuroprotection, TNF-α, succinate dehydrogenase

## Abstract

**Background:** Rosiridin is a compound extracted from *Rhodiola sachalinensis*; water extracts of *Rhodiola* root elicit positive effects on the human central nervous system and improve brain function. They are also thought to be beneficial to one’s health, in addition to being antioxidants. The present study aims to evaluate the anti-Huntington’s effect of rosiridin against 3-nitropropionic acid (3-NPA)-induced Huntington’s disease (HD)-like effects in rats. **Materials and Methods:** The acute toxicity in rats was elucidated to track the conceivable toxicities in the rats. The effectiveness of rosiridin at a dosage of 10 mg/kg was evaluated against several dose administrations of 3-NPA-induced HD-like symptoms in the rats for 22 days. At the end of the study, behavioral parameters were assessed as a hallmark for the cognitive and motor functions in the rats. Similarly, after the behavioral assessment, the animals were sacrificed to obtain a brain tissue homogenate. The prepared homogenate was utilized for the estimation of several biochemical parameters, including oxidative stress (glutathione, catalase, and malondialdehyde), brain-derived neurotrophic factor and succinate dehydrogenase activity, and the glutamate and acetylcholinesterase levels in the brain. Furthermore, inflammatory mediators linked to the occurrence of neuroinflammation in rats were evaluated in the perfused brain tissues. **Results:** The rosiridin-treated group exhibited a significant restoration of behavioral parameters, including in the beam-walk test, latency in falling during the hanging wire test, and percentage of memory retention during the elevated plus-maze test. Further, rosiridin modulated several biochemical parameters, including oxidative stress, pro-inflammatory activity, brain-derived neurotrophic factor, nitrite, and acetylcholinesterase as compared to disease control group that was treated with 3-NPA. **Conclusions:** The current study exhibits the anti-Huntington’s effects of rosiridin in experimental animal models.

## 1. Introduction

Huntington’s disease (HD) is characterized by a complex disorder followed by neurodegeneration. It is most commonly reflected by neuropathology associated with marked motor dysfunctions, such as dystonia and ataxia. This marked pathology occurs due to the process of striatal neuronal degeneration [1,2]. Several studies have suggested that HD-like symptoms arise as a consequence of repeats of trinucleotides, such as cytosine, adenine, and guanine (CAG), on the short arm of the chromosome in the HTT gene. The repeats bring out mutations in the HTT gene, resulting in an unusual polyglutamine expansion, which causes neurodegeneration [3,4,5]. Furthermore, this expansion also results in the aggregation of the HTT protein. The onset of HD is generally between 30 and 50 years of age; however, the chromic nucleotides are repeated, leading to early-onset disease. In addition, the early onset of HD in low-age populations is known as juvenile HD, which is where the onset is at or before 20 years of age with remarkable disturbances in learning and behavior [6].

The gene that encodes for the HTT protein represents fundamental synaptic neuronal functions during the post-embryonic period. Researchers believe that it inhibits apoptosis and protects against toxic mutant HTT. Previous research indicated that mutant proteins can have more or less functionality depending on their mutations [1]. Several areas of the brain contain intranuclear and intracytoplasmic inclusions [7]. It is unknown if the inclusions themselves are pathogenic or if they play a role in disease development. In the striatum, a significant loss of neurons leads to brain atrophy [8,9]. In general, patients have between 36 and 55 CAG repeats on the HTT allele. Currently, mitochondrial dysregulation is being explored in terms of its significant role in the pathology of HD. Brain autopsy studies in HD patients revealed mitochondrial dysfunctions [10,11]. Studies focused on possible therapeutic approaches also explored the process of HTT synthesis, which is controlled by pre-mRNA by using antisense oligonucleotides that contribute to the splicing process through the use of small-molecule splicing modulators on the ribosome by using RNA interference (RNAi) strategies [12]. Recent studies further explored how DNA aptamers can preferentially target mutant huntingtin and modulate a gain of function endowed by the elongated polyglutamine segment [13].

Earlier findings gave insights into some neurotransmitters, such as glutamate and GABA alterations, in HD caused due to mutant huntingtin protein (mHTT). Further, it was also revealed that significant alterations in BDNF, Bax, Bid, NMDA receptors, and intracellular calcium levels were linked with mHTT in HD patients [11,14]. Additionally, studies among rodents evinced a remarkable energy difference between supply and demand, which caused alterations in the normal neuronal activities via mitochondrial dysfunction in HD [10,15]. 

A mitochondrially toxic chemical, 3-nitropropionic acid (3-NPA), irreversibly suppresses the mitochondrial complex II enzyme (succinate dehydrogenase) [16]. The fungal toxin 3-NPA has been proven to cause similar symptoms in both humans and animals. Three-NPA treatment significantly altered the motor functions of rats in terms of their behavioral paradigms (rotarod and locomotor activities) in preclinical studies. Previous data suggested that 3-NPA treatment showed effects that were similar to early and later stages of HD-like symptoms in humans [17,18,19]. Earlier findings explored how treatment with 3-NPA resembles HD pathophysiology, namely, oxidative failure, peroxidation, and particular striatal atrophy [19]. As a result, it has become the gold standard for the clinical induction of HD-like manifestations in rodents.

**Figure 1:**
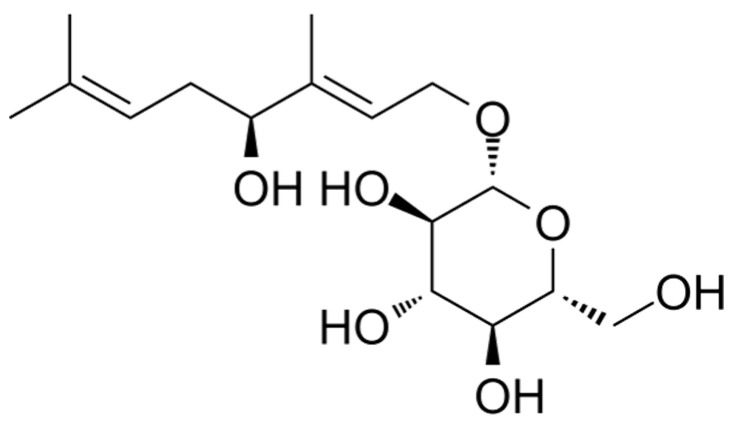
Molecular structure of rosiridin.

Adaptogens were initially classified as chemicals that naturally increase “nonspecific resistance” to stress; this physiology has been linked to a variety of neuroendocrine illnesses [20]. Research on animals and isolated neurons has revealed that adaptogens can exert benefits, such as neuroprotection, anti-fatigue activity, antidepressant activity, anxiolytic benefits, nootropic activity, and CNS stimulation [21,22,23]. Multiple clinical investigations have indicated that adaptogens are effective anti-fatigue agents that boost mental capacity in the face of stress and weariness, including the capacity to endure mental depletion and improve concentration [21]. Several adaptogens have been studied pharmacologically, and many of their effects have been explained by using physiological mechanisms at the molecular level as well [24]. Traditional medicines in the northeast Asian region use the root of root *Rhodiola* species (*Crassulaceae*) as an anti-asthmatic, as a hemorrhage remedy, and as an anti-aging remedy [25,26]. Previous studies have shown that water extracts of *Rhodiola* root elicit positive effects on the human central nervous system and improve brain function [27,28]. They are also thought to be beneficial to one’s health, in addition to being antioxidants [29]. *Rhodiola sachalinensis* produces the chemical compound rosiridin [30], which is shown in Figure 1.

Previously reported data suggested that monoamine oxidase (MAO) activation significantly contributed to the pathogenesis of AD, including the formation of amyloid plaques from Aβ production (Cai, 2014). Since rosiridin inhibits monoamine oxidase A and B, it may be useful in the treatment of despair and early-onset dementia (Panossian and Wikman, 2010; Van Diermen et al., 2009).

## 2. Materials and Methods

### 2.1. Subjects

Adult male Wistar rats (*n* = 6) in a weight range of 150–200 g were acquired and preserved in the laboratory conditions required by the CPCSEA guidelines, which included 40–50% humidity, a temperature of 23 ± 2 °C, and a 12:12 light and dark cycle. Polypropylene cages were employed to contain the rats, and food pellets and water were provided ad libitum. The Institutional Animal Ethics Committee (IAEC No-TRS/PT/022/010) of the Ministry of Environment and Forests, Government of India, New Delhi, provided approvals for the studies on the rodents.

### 2.2. Drugs and Chemicals

The current investigation utilized the following chemicals, which were of analytical grade and from authenticated suppliers: rosiridin, 3-NPA (Sigma-Aldrich, Inc., St. Louis, USA), mannitol, bovine serum albumin, 5, 50-dithiobis-2-nitrobenzoic acid, nitro blue tetrazolium, monosodium phosphate, sodium hydroxide, ethylenediaminetetraacetic acid, hydrogen peroxide, trichloroacetic acid, reduced glutathione, thiobarbituric acid (Hi-Media Laboratories, Mumbai, Pvt, Ltd., India), succinic acid (0.60 M), and potassium ferricyanide (0.03 M) (Trans Asia, Mumbai, India). 

### 2.3. Acute Toxicity Studies

In the present tests, the acute oral toxicity of rosiridin was evaluated according to the OECD guidelines (ANNEX-423 standards). For the toxicity study, rosiridin was orally administered to the rats at the maximum dosage [31,32]. During the evaluation, no rats exhibited mortality or clinical signs of toxicity. Further, these rats did not show a non-significant change in their mean body weight or food and water intake. 

### 2.4. Experimental Design

In the proposed investigation, the experimental design was based on previously reported studies, with trivial modifications. In the grouping, rats were randomly assigned to four distinct groups, with six animals in each group. The grouping was as follows: Group 1 (normal), group 2 (rosiridin *perse*; 10 mg/kg), group 3 (3-NPA), and group 4 (3-NPA + rosiridin treatment of 10 mg/kg, p.o.). In the procedure, at the outset, the animals from all groups were kept for acclimatization for 7 days, as per the standard settings. Consequently, starting on day 8 and for the next 15 days, a single daily intraperitoneal administration of 3-NPA (10 mg/kg) was performed for the significant induction of toxicity in the respective experimental groups [33,34]. From day 8 to day 22, the normal group received saline at 3 mL/kg, and the inducer group, i.e., the 3-NPA control, received oral 0.5% sodium CMC at 3 mL/kg after a 1 h induction of 3-NPA at 10 mg/kg via the intraperitoneal route. Similarly, the test sample group received rosiridin (10 mg/kg p.o. dose) in 0.5% SCMC until day 22 of the drug schedule. During the drug schedule, groups 2, 3, and 4 received 3-NPA at 10 mg/kg (i.p.) 1 h after the aforementioned dose administration. At the end of the experimental schedule, several behavioral paradigms were examined to assess the marked induction of neurophysiological alterations in the rats as a hallmark of HD. At the end of the study, after the last administration of the dosing, the rat brains were removed to perform biochemical evaluations.

### 2.5. Behavioral Estimation

#### 2.5.1. Narrow Beam Walk Assessment

In the behavioral study, balance and prompt motor coordination were assessed in the rats by employing a narrow beam walk test. The basic construction of the apparatus involved an apparatus supplied with a wooden beam with the dimensions of 150 × 4 × 3; it was adjusted to 80 cm above the bottom level and supported by wooden pillars, which secured both ends of the beam. The animals were trained (for 2 min) to traverse this wooden beam from end to end. In the procedure, the total time required to pass across the dedicated beam was considered for the assessment [33]. 

#### 2.5.2. Hanging-Wire Test

The hanging-wire test was carried out to assess the gripping strength of the rats. The specification included a 80 cm × 2 mm wire tied to both sides of a table with an elevation of 40 cm above the floor. As the hallmark of grip strength, the latency of falling (s) was assessed while the rats were hanging onto the rope, with a 60 s cut-off period for the evaluation [32,35].

#### 2.5.3. Elevated Plus-Maze Test

In the elevated plus-maze (EPM) testing paradigm, spatial memory was assessed as a hallmark of learning and memory in the experimental animal model with chemically induced HD-like symptoms [33]. In the procedure of the EPM test, the rodents explored to evaluate areas with open spaces in order for their spatial memory to be assessed. The EPM specification included an apparatus made of a wooden material with two arms (open and closed) facing each other. The construction of both arms included sections with specifications of 50 × 10 cm and 50 × 10 × 42 cm. Both arms were connected to a central intersecting platform (10 × 10 cm). The EPM was elevated 50 cm above the floor. Before testing, each rat was trained via exposure to each arm. The cut-off time for each trial was set to 90 s, during which the time required by each rat to reach the closed arm from the open arm—with all 4 of its limbs in the closed arm—was taken to be the transfer latency (TL). Rats who did not reach the closed arm during the cut-off period were gently guided to reach to closed arm within the specified time limit. An addition 2 min were given to the animals to explore the EPM before returning to their home cages during the acquisition trial. After 24 h, the TL was recorded as a component of spatial memory retrieval for induvial rats in their respective groups. At the end of the study, before proceeding to the biochemical estimation, the animals were subjected to a retrieval testing paradigm, during which a shorter TL in the rats was considered a hallmark for spatial memory.

### 2.6. Biochemical Estimation

#### 2.6.1. Brain Homogenate

At the end of the treatment schedule and after the behavioral assessments, each rat was euthanized to isolate the brain for the preparation of a brain tissue homogenate. The whole isolated brain was rinsed with ice-cold isotonic 0.9% NaCl saline solution. Immediately, after the rinsed brain was put forward for homogenization (Remi, Mumbai, India) in the presence of a buffer solution that was utilized for separation, it was maintained at a pH of 7.4 and in an ice-cold form. The buffer used for homogenization was composed of EGTA, mannitol, BSA, sucrose, and HEPES in all required concentrations [36]. Afterward, the brain homogenate was subjected to centrifugation at 4 °C for 15 min with a force of 13,000× *g*. 

#### 2.6.2. Brain Biochemical Parameters

In the present investigation, different brain biochemical was assessments were performed to evaluate the involvement of several biomarkers in the pathogenesis of HD. The standard estimation (Transasia Biomedical Ltd., Mumbai, India)—as per previously reported studies—was employed for the measurement of brain glutathione (GSH) [37], catalase (CAT) [38], malonaldehyde (MDA) [39], and pro-inflammatory TNF-α and IL-1β [40]. 

#### 2.6.3. Estimation of Brain-Derived Neurotrophic Factor (BDNF) Activity

Commercially available ELISA kits (Thermo-Fisher Scientific, Pune, India) were employed as per the manufacturer’s recommendations and a previously reported methodology for the estimation of BDNF activity in the brain homogenate [41]. 

#### 2.6.4. Estimation of Succinate Dehydrogenase (SDH) Activity

The activity of an important mitochondrial biomarker, SDH, was estimated by employing a well-established spectrophotometry technique [42]. The methodology consisted of the oxidation of succinate via potassium ferricyanide. The solution also contained succinic acid, BSA, and a buffer (Na^+^ K^+^ PO_4_^3−^) in the necessary concentrations as per previously reported studies, and the solution was maintained at a pH of 7.80 throughout the procedure. The mitochondrial sample was added and subjected to spectrophotometric analysis at a wavelength of λ_max_ = 420 nm for 2 min. The SDH activity (nmol succinate oxidized/min/mg protein) was measured.

#### 2.6.5. Estimation of Glutamate

The glutamate estimation was performed via a suitable spectrophotometric analysis. In the procedure, column ovens, rheodine injectors, quaternary pumps, and ion traps for UV detection were utilized to set the HPLC instruments at different wavelengths. The sample injection was performed via a column (AQUA, 150 mm 5 μ C18) (Phenomenex, Hyderabad, India). During the analysis of the sample, a standard laboratory environment was maintained, which included a rate of 1.5 mL/min (UV 190 nm), a mobile-phase (20 nM) phosphate buffer, and a pH of 2.5. Solid-phase extraction was rapidly performed to eliminate trace elements and lipids that occurred in the sample. 

Glutamate was separated after 12 min of initial run time. A comparison was made with a standard chromatogram (Sigma-Aldrich, St. Louis, USA) to locate the position of glutamate in the sample. Finally, the concentration of ng of glutamate per gram of brain tissue was calculated [43]. The precolumn PITC derivatization method was employed to identify free amino acid neurotransmitters as per previously reported studies [44]. 

#### 2.6.6. Nitrite Content Estimation

The procedure for the estimation of nitrite content was based on previously reported data [45]. Precisely, the estimation of the sample included a test tube with the required amount of testing solution (0.10 mL), standards, copper cadmium alloy, and buffer solution (carbonic acid) at a pH of 9.0. The entire solution was subjected to incubation for 1 h. The necessary amounts of NaOH and ZnSo4 were added to the solution, and it was subjected to centrifugation at 4000× *g* for 10 min. After centrifugation, the supernatant was separated and mixed with 0.05 mL of Griess reagent. The final mixture was then subjected to 30 min of incubation in a dark place, and then put forward for a spectrophotometric evaluation at λ_max_ = 548 nm. A standard curve was plotted, and the total nitrite content in the sample was compared and presented as μM per mg of brain protein. 

#### 2.6.7. Evaluation of Acetylcholinesterase (AchE) Levels

The AchE expression was quantified by using the technique described by ELLMAN [37]. The AchE activity was measured in M/mg protein 59.

### 2.7. Statistical Analysis

We utilized Prism 5 for Windows to analyze the data in this study (version 5.02). The findings were summarized as the mean ± SEM. To examine the amount of significance and illustrate the variation between the parameters within each category, a one-way analysis of variance (ANOVA) was performed, which was preceded by Dunnett’s post hoc parametric test. Only *p* values under 0.05 were considered statistically significant.

## 3. Results

### 3.1. Acute Toxicity Study

In the 14-day acute toxicity period, there were no mortalities or clinical features of abnormalities. In light of the findings of the acute oral toxicity investigation, we decided to conduct the main investigation with 10 mg/kg of rosiridin.

### 3.2. Behavioral Assessment

**Figure 2:**
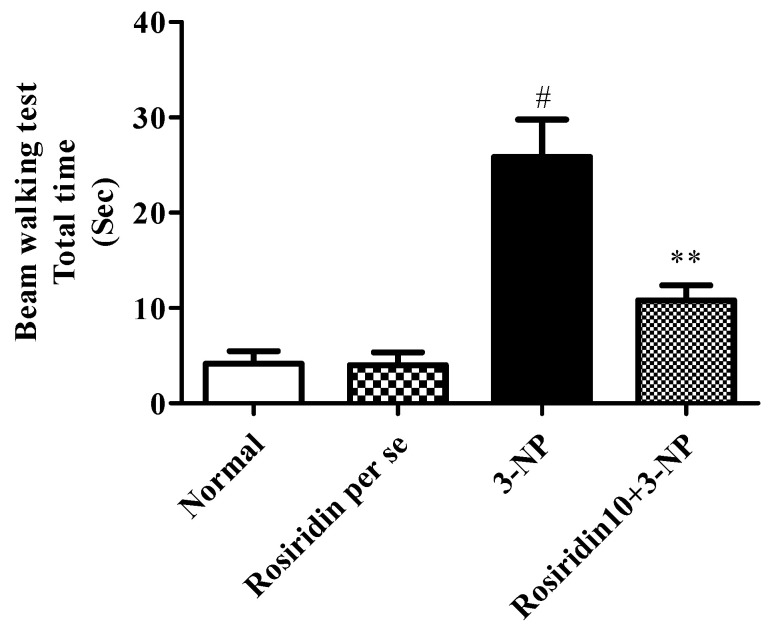
Effect of rosiridin on the narrow beam walk assessment. Values are expressed as the mean ± SEM (n = 6). Values are statistically significant at *p* < 0.05 (one-way ANOVA followed by Dunnett’s test). Express the data (^#^ *p* < 0.05) saline vs. negative control group; (** *p* < 0.01) negative control group vs. treatment group.

Figure 2 describes the influence of rosiridin on the behavioral assessment of the rats on the narrow beam walk while experiencing the 3-NPA-induced Huntington’s-like symptoms, revealing that the rats in the 3-NPA control group took a significantly longer time to walk across the wooden platform (# *p* < 0.001) compared to the normal group. The normal memory function of the rosiridin-treated (10 mg/kg) group was restored, as shown by the significant decrease in the time required to walk across the beam compared to the 3-NPA control group (** *p* < 0.01). However, the one-way ANOVA followed by Dunnett’s post hoc parametric test analysis showed that narrow beam walk test in rats treated with the rosiridin per se or with vehicle was comparable.

#### 3.2.1. Effect of Rosiridin on the Hanging-Wire Test

**Figure 3:**
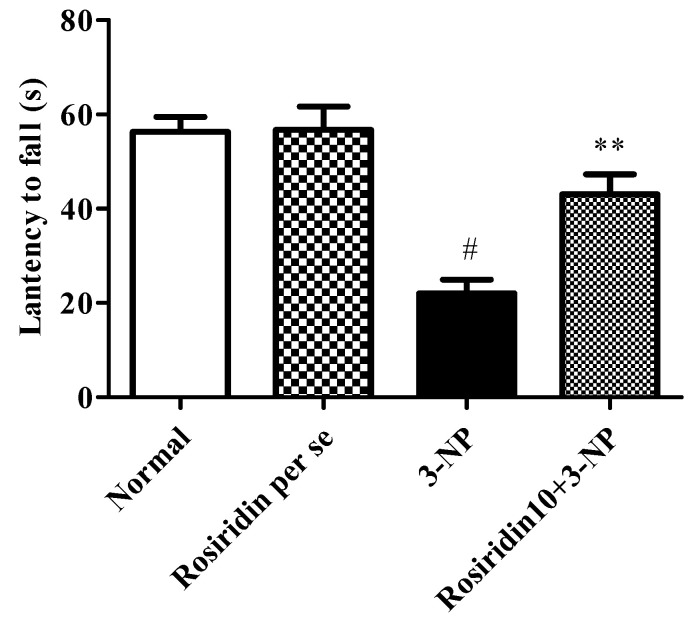
Effect of rosiridin on the hanging-wire test. Values are expressed as the mean ± SEM (n = 6). Values are statistically significant at *p* < 0.05 (one-way ANOVA followed by Dunnett’s test). Express the data (^#^ *p* < 0.05) saline vs. negative control group; (** *p* < 0.01) negative control group vs. treatment group.

Figure 3 depicts the impact of rosiridin on the behavioral assessment in the hanging-wire test as a hallmark of skeletal muscle strength in the rats experiencing 3-NPA-induced Huntington’s-like symptoms. In the assessment, the 3-NPA control group exhibited a significant decline in latency of fall time (# *p* < 0.001) during the hanging-wire test compared to the normal group. Similarly, the one-way ANOVA and Dunnett’s post hoc parametric test analysis revealed that the rosiridin (10 mg/kg) treatment group exhibited restored normal gripping strength with an improvement in the time required for the fall compared to the 3-NPA control group (** *p* <0.01). However, rosiridin per se or with vehicle was comparable during the hanging-wire test according to the parameters in the rats.

#### 3.2.2. Effect of Rosiridin on the Elevated Plus-Maze Test

**Figure 4:**
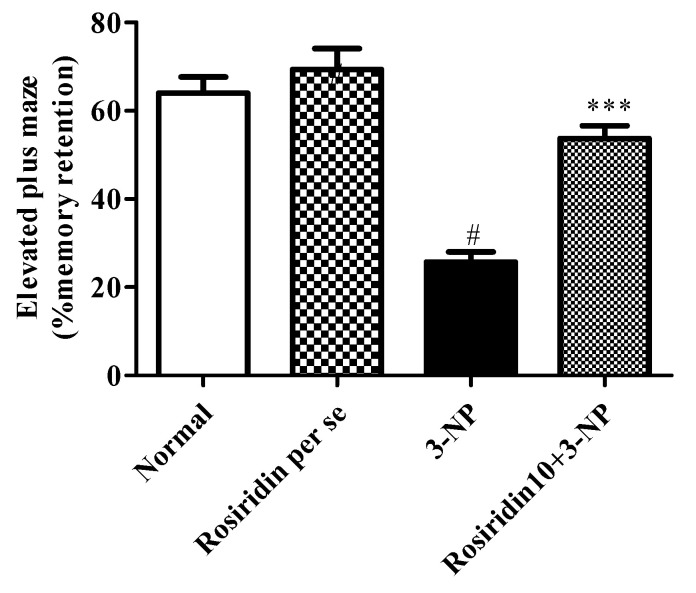
Effect of rosiridin on the elevated plus-maze test. Values are expressed as the mean ± SEM (n = 6). Values are statistically significant at *p* < 0.05 (one-way ANOVA followed by Dunnett’s test). Express the data (^#^ *p* < 0.05) saline vs. negative control group; (*** *p* < 0.001) negative control group vs. treatment group.

Figure 4 portrays the influence of rosiridin on the elevated plus-maze test as a hallmark of spatial memory in the experimental animal model that was subjected to 3-NPA-induced Huntington’s-like symptoms. The evaluation revealed that the 3-NPA control group exhibited a significant decline in spatial memory retention (# *p* < 0.001) during the EPM assessment compared to the normal group. Additionally, the one-way ANOVA and Dunnett’s post hoc parametric test analysis revealed that the rosiridin (10 mg/kg) treatment group experienced restored normal memory function in comparison with the 3-NPA control group (*** *p* < 0.001). However, the EPM test in rats treated with the rosiridin per se or with vehicle was comparable.

### 3.3. Biochemical Estimation

#### 3.3.1. Effect of Rosiridin on Oxidative Stress Parameters

**Figure 5:**
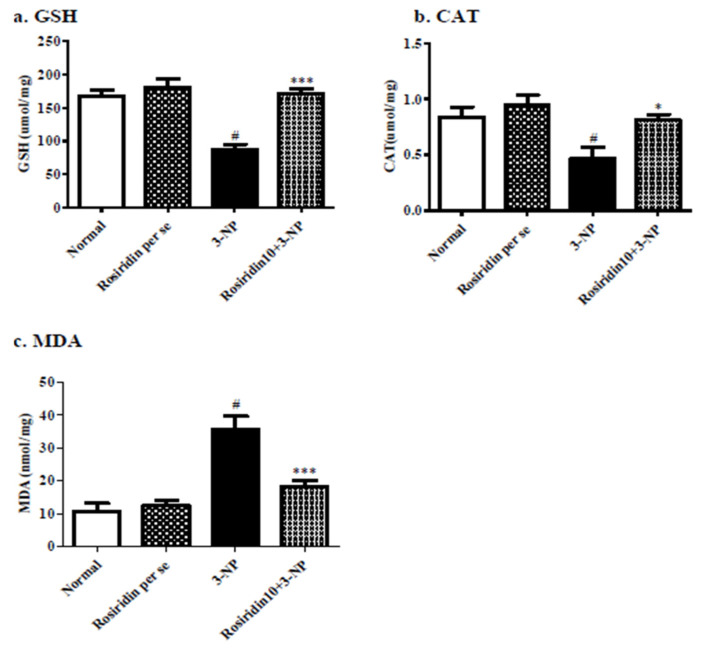
Effect of rosiridin on oxidative stress parameters: (**a**) GSH, (**b**) CAT, and (**c**) MDA. Values are expressed as the mean ± SEM (n = 6). Values are statistically significant at *p* < 0.05 (one-way ANOVA followed by Dunnett’s test). Express the data (^#^ *p* < 0.05) saline vs. negative control group; (*** *p* < 0.001; * *p* < 0.05) negative control group vs. treatment group.

Figure 5 ((a) GSH, (b) CAT, (c) MDA) illustrates the effect of rosiridin on the redox imbalance parameters in rats with 3-NPA-induced Huntington’s-like symptoms. When compared to the rats in the normal group, the rats in the 3-NPA-induced control group had a substantial (# *p* < 0.001) increase in MDA activity-a key biomarker for ROS. Similarly, in another set of experiments, the 3-NPA-induced rats showed an extensive reduction in oxidative biomarkers such as GSH and CAT in their brain tissue (*** *p* < 0.001) in comparison with the normal group. In addition, one-way ANOVA and Dunnett’s post hoc parametric test analysis revealed that daily rosiridin (10 mg/kg) treatment for 15 days exceedingly reduced the MDA activity (*** *p* < 0.001) and resulted in a remarkable restoration of GSH (** *p* < 0.01) and CAT (* *p* < 0.05) activities compared to those in the 3-NPA control group. Furthermore, the oxidative stress level of rats treated with the rosiridin per se or with vehicle was comparable.

#### 3.3.2. Effect of Rosiridin on Proinflammatory Biomarkers

**Figure 6:**
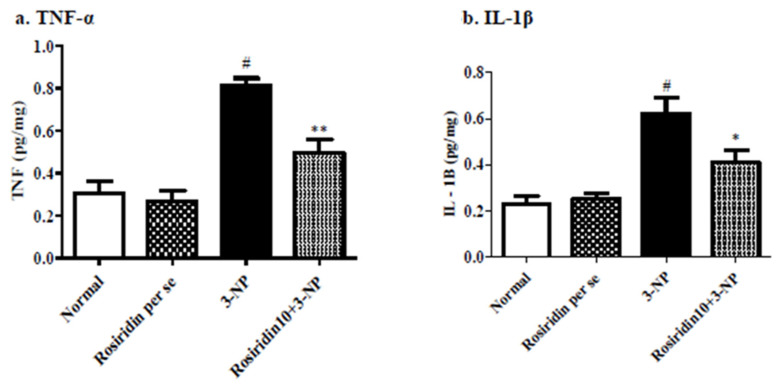
Effect of rosiridin on proinflammatory biomarkers ((**a**) TNF-α and (**b**) IL-1β). Values are expressed as the mean ± SEM (n = 6). Values are statistically significant at *p* < 0.05 (one-way ANOVA followed by Dunnett’s test). Express the data (^#^ *p* < 0.05) saline vs. negative control group; (** *p* < 0.01; * *p* < 0.05) negative control group vs. treatment group.

Figure 6 ((a) TNF-α, (b) IL-1β) exemplifies the effect of rosiridin on proinflammatory mediators in rats with 3-NPA-induced Huntington’s-like symptoms. In the analysis, the 3-NPA-induced control group showed a remarkable elevation in certain pro-inflammatory mediator levels, such as the TNF-α (# *p* < 0.001) and IL-1β (*** *p* < 0.001) levels, compared to the normal group. Similarly, one-way ANOVA and Dunnett’s post hoc parametric test analysis revealed that the group of animals that received a daily defined dose of rosiridin (10 mg/kg) before 3-NPA administration experienced a significant restoration of proinflammatory biomarkers, such as TNF-α (** *p* < 0.01) and IL-1β (* *p* < 0.05), in the brain. Furthermore, proinflammatory mediators in the rats treated with the rosiridin per se or with vehicle was comparable.

#### 3.3.3. Effect of Rosiridin on Brain-Derived Neurotrophic Factor (BDNF) Activity

**Figure 7:**
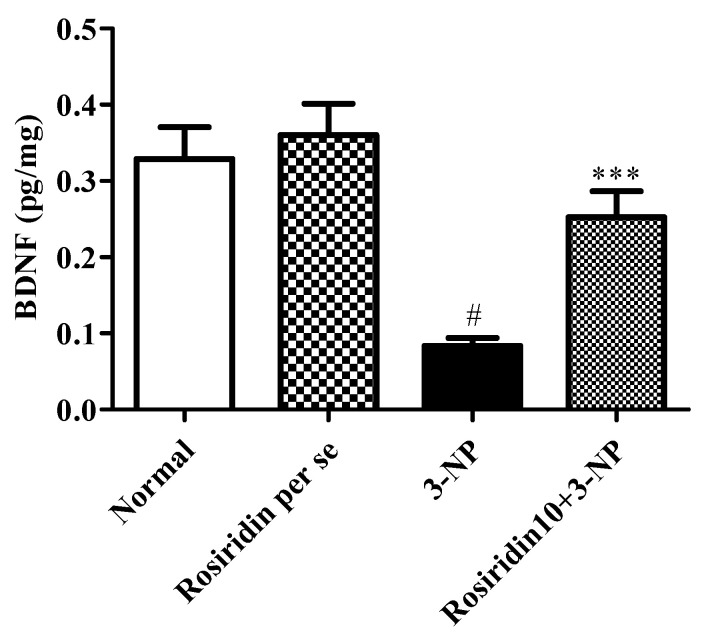
Effect of rosiridin on brain-derived neurotrophic factor (BDNF) activity. Values are expressed as the mean ± SEM (n = 6). Values are statistically significant at *p* < 0.05 (one-way ANOVA followed by Dunnett’s test). Express the data (^#^ *p* < 0.05) saline vs. negative control group; (*** *p* < 0.001) negative control group vs. treatment group.

Figure 7 illustrates the brain BDNF activity in rats with 3-NPA-induced Huntington’s-disease-like effects and the role of rosiridin in modifying this activity in the rats. In the current investigation, we discovered that the rats in the 3-NPA control group exhibited a significant diminution in their brain BDNF activity (# *p* < 0.001), which showed the contribution of 3-NPA to the denaturation of the brain and neuronal tissue. In the present investigation, one-way ANOVA followed by Dunnett’s post hoc parametric test analysis revealed that daily rosiridin (10 mg/kg) doses for 15 days significantly restored the normal brain function through the modulation of BDNF activity (*** *p* < 0.001) in rats when compared to the 3-NPA control group. Furthermore, BDNF activity in the rats treated with the rosiridin per se or with vehicle was comparable.

#### 3.3.4. Effect of Rosiridin on Succinate Dehydrogenase (SDH) Activity

**Figure 8:**
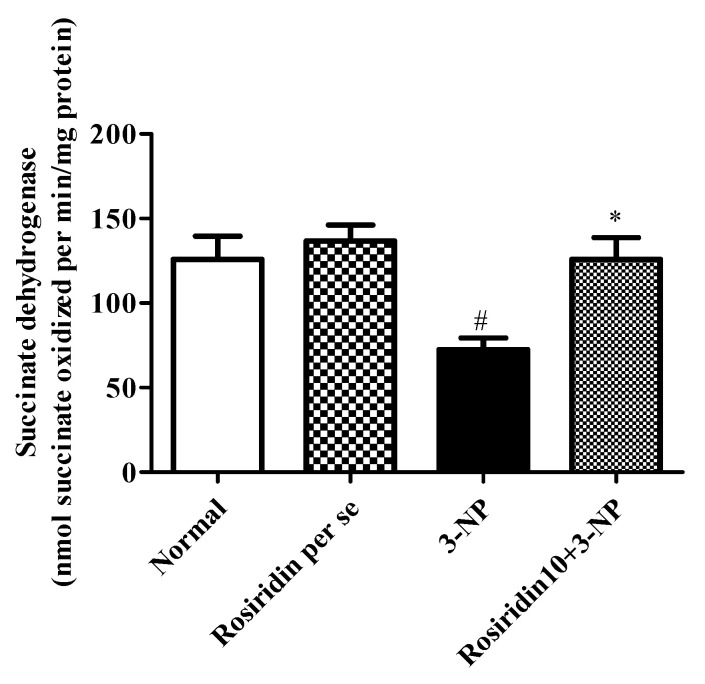
Effect of rosiridin on succinate dehydrogenase (SDH) activity. Values are expressed as the mean ± SEM (n = 6). Values are statistically significant at *p* < 0.05 (one-way ANOVA followed by Dunnett’s test). Express the data (^#^ *p* < 0.05) saline vs. negative control group; (* *p* < 0.05) negative control group vs. treatment group.

Figure 8 illustrates the succinate dehydrogenase activity in rats with 3-nitropropionic-acid-induced Huntington’s-disease-like effects and the role of rosiridin in modifying this activity in the rats. The findings revealed that the rats in the 3-NPA-control group showed a significant attenuation in their succinate dehydrogenase activity (# *p* < 0.001) as hallmarks injuries in the brain and neuronal tissue. Furthermore, one-way ANOVA followed by Dunnett’s post hoc parametric test analysis revealed that daily rosiridin (10 mg/kg) doses for 15 days before 3-NPA administration remarkably restored the normal brain function through the modulation of succinate dehydrogenase activity (*** *p* < 0.001). Furthermore, succinate dehydrogenase activity in the rats treated with the rosiridin per se or with vehicle was comparable.

#### 3.3.5. Effect of Rosiridin on Glutamate Activity

**Figure 9:**
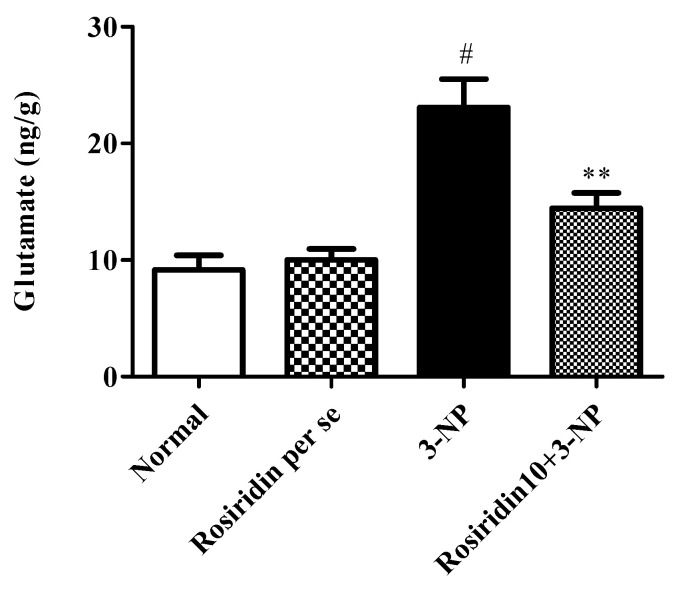
Effect of rosiridin on glutamate activity. Values are expressed as the mean ± SEM (n = 6). Values are statistically significant at *p* < 0.05 (one-way ANOVA followed by Dunnett’s test). Express the data (^#^ *p* < 0.05) saline vs. negative control group; (** *p* < 0.01) negative control group vs. treatment group.

Figure 9 illustrates the effect of glutamate expression in rats with 3-NPA-induced Huntington’s-disease-like symptoms and the role of rosiridin in altering this activity in the rats. The current investigation discovered that the rats in the 3-NPA control group had an overexpression of glutamate compared to the normal group (# *p* < 0.001). Furthermore, one-way ANOVA followed by Dunnett’s post hoc parametric test analysis revealed that daily rosiridin (10 mg/kg) doses for 15 days significantly modulated the glutamate expression compared to that in the 3-NPA control group (** *p* < 0.01). Additionally, glutamate activity in the rats treated with the rosiridin per se or with vehicle was comparable.

#### 3.3.6. Effect of Rosiridin on Nitrite Content

**Figure 10:**
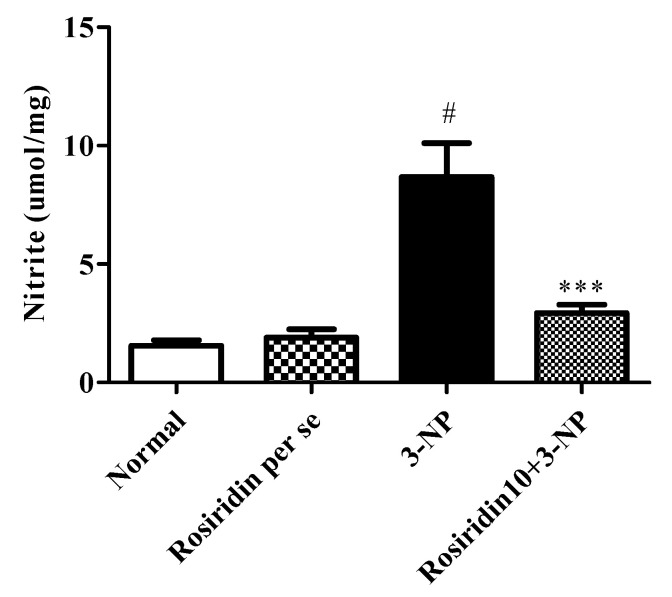
Effect of rosiridin on nitrite content. Values are expressed as the mean ± SEM (n = 6). Values are statistically significant at *p* < 0.05 (one-way ANOVA followed by Dunnett’s test). Express the data (^#^ *p* < 0.05) saline vs. negative control group; (*** *p* < 0.001) negative control group vs. treatment group.

Figure 10 demonstrates the effect of rosiridin on nitrite content in rats with 3-NPA-induced Huntington’s-disease-like effects. The present study found that the animals in the 3-NPA control group had significantly elevated levels of nitrite compared to those of the normal group (# *p* < 0.001). Further, one-way ANOVA followed by Dunnett’s post hoc parametric test analysis revealed that daily rosiridin (10 mg/kg) doses for 15 days pointedly modulated the nitrite levels compared to those of the 3-NPA control group (*** *p* < 0.001). Additionally, nitrate levels in the rats treated with the rosiridin per se or with vehicle was comparable.

#### 3.3.7. Effect of Rosiridin on Acetylcholinesterase (AchE) Levels

**Figure 11:**
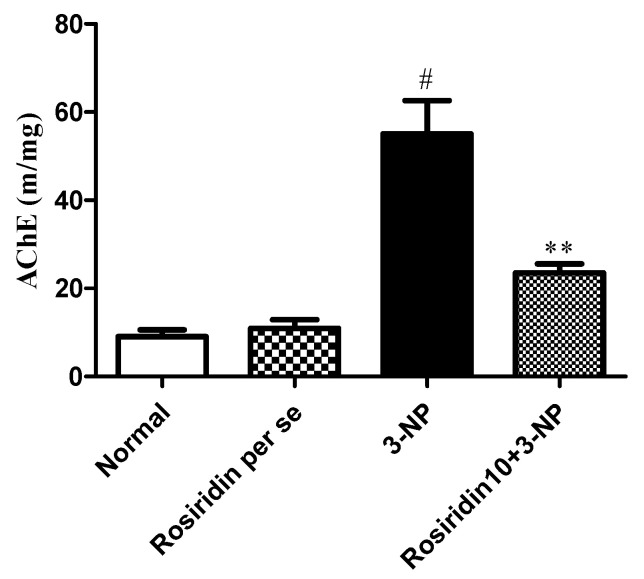
Effect of rosiridin on acetylcholinesterase (AchE) levels. Values are expressed as the mean ± SEM (n = 6). Values are statistically significant at *p* < 0.05 (one-way ANOVA followed by Dunnett’s test). Express the data (^#^ *p* < 0.05) saline vs. negative control group; (** *p* < 0.01) negative control group vs. treatment group.

Figure 11 illustrates the effect of rosiridin on AchE levels on rats with 3-NPA-induced Huntington’s-disease-like effects. We found that the animals in the 3-NPA control group had significantly elevated levels of AchE compared to those in the normal group (# *p* < 0.001). Further, one-way ANOVA followed by Dunnett’s post hoc parametric test analysis showed that daily rosiridin (10 mg/kg) doses for 15 days remarkably restored the normal AchE levels in comparison with the levels in the 3-NPA control group (** *p* < 0.01). Additionally, AchE levels in the rats treated with the rosiridin per se or with vehicle was comparable.

## 4. Discussion

HD is clinically diagnosed in patients with marked behavioral and locomotor alterations, followed by a parental DNA analysis to determine the possibility of transmission to progeny. In individuals that are at high risk for HD, a preventative diagnostic test for the existence of carrier genes may be conducted. The condition has no known cure as of yet, and affected people are completely reliant on their caretakers as the disease advances. As a result, a treatment regimen can improve quality of life while reducing problems associated with HD [4,25,26]. Studies have also explored clinical manifestations of HD, including motor, cognitive, and psychological impairments. These manifestations are more commonly observed at an early age and could lead to the death of the individual [46]. In the present investigation, we explored the possible neuroprotection offered by rosiridin against HD-like symptoms in experimental animal models. 

Previously reported studies suggested the significant roles of several antioxidants that modulate the pathology and manifestations associated with HD. Furthermore, studies also highlighted the significance of antioxidants of several natural origins in the management of HD-like symptoms in experimental animal models [47]. At the onset of our investigation, acute toxicity tests were performed to examine any toxicities linked with rosiridin. According to the findings, rosiridin with a dose of 10 mg/kg was proven to be safe in the rats, with no observable clinical manifestations. Previous data suggested the involvement of 3-NPA as a potential mitochondrially toxic component known to result in HD-like symptoms in experimental animal models [48]. 

In the present investigation, it was observed that 3-NPA potentially caused HD-like symptoms in rats. The clinical correlation between free radical formation and the mitochondrial dysregulations associated with HD-like symptoms is well demonstrated in the available literature [49,50]. The literature has also indicated that 3-NPA administration causes significant alterations in the normal motor functions of experimental animals. Similarly, in our findings, we observed significant motor alterations in the rodents during the behavioral assessments. The results of the behavioral tests demonstrated the remarkable alterations in the motor functions and gripping strength induced by 3-NPA in the animals. Surprisingly, pre-treatment with rosiridin (10 mg/kg) prevented motor deficits and loss of grip strength in 3-NPA-treated rats.

The EPM test showed that 3-NPA devastatingly affected the cognitive functions of the animals. The molecular mechanism involved in 3-NPA-induced CNS pathology mainly functions through injuries that occur at the different brain levels, such as the cortex, hippocampus, and striatum. Earlier data suggested that, in HD, marked dysregulations occur at the mitochondrial level, and elevated oxidative stress imparts significant memory and cognitive impairment [35,51]. In our finding, we observed that rosiridin efficiently protected spatial memory in rats exposed to 3-NPA. 

In the present study, in addition to the behavioral assessments, we performed several biochemical estimations to explicate alterations that were linked with HD. Previously reported studies pointed out the influence of several biochemical parameters that contributed to the strengthening of the pathogenesis of HD [48,52,53]. The biochemical estimations mainly involved the assessment of oxidative stress parameters, estimation of proinflammatory mediators, quantification of several brain enzymatic activities (AchE and succinate dehydrogenase), and evaluation of the expression of several amines. Additionally, previous data showed marked alterations in the GSH, SOD, CAT, LPO, GR, and nitrite levels in the brain and neuronal tissues; these alterations were associated with HD [48,52]. We found that the 3-NPA-treated animals exhibited a significant decline in the levels of CAT and GSH and showed elevated MDA and nitrite content in the brain. The treatment with rosiridin for 15 days significantly restored the oxidative stress biomarkers and nitrite levels. 

Earlier studies suggested that BDNF levels are lower in animal models of HD [54]. The significant alterations in BDNF are associated with mRNA expression, HTTT dysregulation, and overproduction of reactive oxygen species [55]. Similarly, we found that animals treated with 3-NPA exhibited significant alterations in the BDNF. Further, rosiridin restored the normal levels of BDNF after 3-NPA-induced neurotoxicity in rats. Earlier data also elucidated the significant irregularities that connect several neurotransmitters with HD. Importantly, several neurotransmitters, such as serotonin, dopamine, glutamate, and GABA, have a significant role in HD pathology [14,51,56,57]. We observed the levels of glutamate in the brain tissue homogenate, and we found that 3-NPA intensely altered the glutamate levels. In contrast, the rosiridin treatment significantly restored the normal levels in the rats.

These pieces of evidence also suggest the critical aspects of inflammation in neurodegenerative diseases. Numerous data suggested that proinflammatory biomarkers, such as TNF-α, IL-1β, and MPO, are an important hallmark for the pathology of neurodegeneration [58,59]. In the present study, we observed that treatment with rosiridin provided neuroprotective effects by preventing oxidative stress and AchE, modulating the succinate dehydrogenase, nitrite, and BDNF levels, and restoring the inhibited antioxidant defense system. Further, in the present investigation, we discovered that 3-NPA administration in the rats noticeably altered the proinflammatory biomarkers, which was efficiently counteracted by treatment with rosiridin for 15 days, suggesting its anti-neuroinflammatory potential. 

## 5. Conclusions

The significant involvement of biological factors, including mitochondrial dysfunction, elevated the oxidative stress and altered the expression of several biogenic proteins in the pathogenesis of HD. The present study revealed the potential neuroprotective role of rosiridin through its effects on oxidative stress, the modulation of AchE, and the mitochondrial enzyme complex against 3-NPA-induced HD-like symptoms in rats. Furthermore, the clinical effectiveness of rosiridin must be promptly addressed in order for its possible therapeutic applications in the management of neurodegenerative diseases, with a strong focus on HD, to be predicted. 

## Data Availability

Not applicable.
